# 4-Pyridinio-1,4-Dihydropyridines as Calcium Ion Transport Modulators: Antagonist, Agonist, and Dual Action

**DOI:** 10.1155/2020/2075815

**Published:** 2020-03-27

**Authors:** Ilona Domracheva, Iveta Kanepe-Lapsa, Reinis Vilskersts, Imanta Bruvere, Egils Bisenieks, Astrida Velena, Baiba Turovska, Gunars Duburs

**Affiliations:** ^1^Latvian Institute of Organic Synthesis, Group of Experimental Chemotherapy, Aizkraukles Iela 21, Riga, Latvia LV-1006; ^2^Latvian Institute of Organic Synthesis, Laboratory of Pharmaceutical Pharmacology, Aizkraukles Iela 21, Riga, Latvia LV-1006; ^3^Latvian Institute of Organic Synthesis, Laboratory of Membrane Active Compounds and β-Diketones, Aizkraukles Iela 21, Riga, Latvia LV-1006; ^4^Latvian Institute of Organic Synthesis, Laboratory of Physical-Organic Chemistry, Aizkraukles Iela 21, Riga, Latvia LV-1006

## Abstract

A set of six new 4-pyridinio-1,4-dihydropyridine (1,4-DHP) compounds has been synthesized. The calcium channel modulating activity of these compounds was evaluated in an aorta vascular smooth muscle cell line (A7R5), in an isolated rat aortic ring model, and in human neuroblastoma cell lines (SH-SY5Y). The antagonistic effect of these 1,4-DHP was tested by modulating the impact of carbachol-dependent mobilization of intracellular Ca^2+^ in SH-SY5Y cells. The intracellular free Ca^2+^ concentration was measured in confluent monolayers of SH-SY5Y cells and A7R5 cells with the Ca^2+^-sensitive fluorescent indicator Fluo-4 NW. Only four compounds showed calcium channel blocking activity in SH-SY5Y and A7R5 cells as well as in the aortic ring model. Among them, compound 3 was the most active calcium channel antagonist, which had 3 times higher activity on carbachol-activated SH-SY5Y cells than amlodipine. Two of the compounds were inactive. Compound 4 had 9 times higher calcium agonist activity than the classic DHP calcium agonist Bay K8644. The intracellular mechanism for the action of compound 4 using inhibitor analysis was elucidated. Nicotinic as well as muscarinic receptors were not involved. Sarcoplasmic reticulum (ER) Ca^2+^ (SERCA) stores were not affected. Ryanodine receptors (RyRs), another class of intracellular Ca^2+^ releasing channels, participated in the agonist response evoked by compound 4. The electrooxidation data suggest that the studied compounds could serve as antioxidants in OS.

## 1. Introduction

The dihydropyridines (DHPs), especially 1,4-DHP, are a class of polyfunctional (pleiotropic) redox-active organic compounds.

1,4-DHP is an analogue of 1,4-dihydronicotinamide and model compounds of redox-coenzymes NAD(P)H, which participates in redox reactions and can act as deactivators (quenchers) of reactive oxygen species (ROS) and reactive nitrogen species (RNS) [1].

1,4-DHP is widely used as pharmaceuticals because of their cardiac inotropic and vasomotor effects. Numerous members of this class are important commercial cardioprotectors, vasodilators, and calcium antagonists [2, 3], modulating not only metabolic pathways that involve Ca^2+^ ions [2], including voltage-operating (VOC), receptor-operating (ROC), and store-operating (SOC) calcium channels, but also acting on other targets: alpha-/beta-adrenoreceptors, potassium channels [2], as well as being effectors of oxidative stress (OS) [1, 4]. Homeostasis of Ca^2+^ ions is important for metabolic functions in living cells [5]. Under the conditions of OS, this homeostasis is disrupted. Therefore, DHP compounds that modulate the transport of Ca^2+^ ions [6] may indirectly protect against OS lesions in vascular, cardiac, and other tissues.

DHP modulate Ca^2+^ transport either as blockers (e.g., nifedipine, nimodipine, nitrendipine, and amlodipine) [7] or as promoters (e.g., calcium agonists Вау K8644, CGP28392, and (+)-PN-202-791) [5, 6]. Stereoisomers of DHP may exhibit the opposite effects. For example, (+)-PN-202-791 is calcium аgonist, while (-)-PN-202-791 acts as the аntagonist [8, 9]. Different effects have been observed for stereoisomers of Вау K8644 [10]. In the same experimental model, low concentrations of DHP acting as calcium antagonists (nifedipine, nitrendipine, and nicardipine) could express agonist (positive inotropic) effect [11], while high concentrations of the same agonist compounds exerted аntagonist effect [12]. Compounds with the aforementioned properties have been referred to as “dual-acting agents” (cardioselective calcium channel agonist-smooth muscle selective calcium channel antagonist, depending on the cell type) and have been also classified as “third-generation DHP” [13]. The concentration effects (high versus low doses) in the expression of agonist/antagonist properties have not yet been sufficiently explored.

The nature of the binding sites for antagonists and agonists is variously defined and not fully understood. So, one high affinity binding site for both antagonists and agonists is proposed. This idea has been confirmed by binding and pharmacological experiments, which showed a competition between DHP Ca^2+^ channel antagonists and agonists (as reviewed by Glossmann et al. [14] and Williams et al. [15]). A model postulating one site for agonists and another for antagonists is based on a cooperative interaction between DHP agonists and antagonists, which was demonstrated in cardiac cells [5, 16]. Thus, the number of sites and the interactions between the effects of different DHP remain unclear [17]. It was found in other studies that the high affinity site was either stimulatory or inhibitory for Ca^2+^ channels, depending upon the membrane potential, and that the low affinity site was stimulatory [7]. The DHP derivative CGP 28861 can convert the DHP Ca^2+^-channel receptor from an antagonistic site into an agonistic one. The molecular mechanism responsible for the observed effect is unknown [17].

DHP acting as Ca^2+^ antagonist exhibit the vasorelaxant action, useful for many clinical indications. However, their negative effects on cardiac contractility are still of a great concern especially for patients with heart failure.

A more complete understanding of the occurrence and mechanisms of antagonistic versus agonistic or antagonistic/agonistic effects of DHP could prove useful for new drug design. Dual-acting DHP compounds, such as smooth muscle calcium channel antagonist/cardiac muscle calcium channel agonist, may provide benefits particularly for patients with compromised cardiac contractility [3]. Compound AK-2-38, which is a C-4 2-pyridinyl DHP [18, 19], is a close analogue of 4-pyridinio-DHP. Although it exhibited twice as high potency as nifedipine on smooth muscle, its dosage range that inhibited smooth muscle contraction (i.e., antagonistic activity) resulted in partial agonism on cardiac muscle.

High level Ca^2+^ channel blocking activity was found in the studies of novel derivatives of the 3^rd^ generation DHP Ca^2+^ antagonist amlodipine on SH-SY5Y cells [20].

In the present study, the antagonism/agonism of six new 4-pyridinio-1,4-DHP (*alias* 4-pyridinium-1,4-DHP) derivatives (see Table 1) against L-type Ca^2+^ channels was detected using three model systems: (1) SH-SY5Y human neuroblastoma cells (having two types of Ca^2+^ channels—L-type and N-(T-) type), (2) aorta A7R5 cells [21] (having only L-type calcium channels, as recently reported in Saddala et al. [22]), and (3) isolated rat aorta ring.

The antagonist effect was compared to that of amlodipine, and agonist properties were compared to Bay K8644. Amlodipine as a 3^rd^ generation Ca^2+^ antagonist and pleiotropic compound has been described as modulator of oxidative stress, having antioxidant and antiradical activity (see as referenced in Vitolina et al. [23]).

A dual antagonist/agonist effect of one of DHPs (compound 4 (code No. IB-113), see Tables 1 and 2) on calcium channels was observed in the present study.

Some 4-pyridinio-1,4-dihydropyridine derivatives have been claimed as new agoallosteric modulators of adenosine A2A receptor [24].

Antiviral activity of analogues of the presented six compounds has been described [25]. Besides, close analogues of the mentioned compounds were found to possess cell growth modulator properties [26].

In our research, the involvement of muscarinic, nicotinic, and ryanodine receptors, as well as endoplasmic reticulum Ca^2+^ transport systems, in the intracellular action of 4-pyridinio-1,4-dihydropyridines was studied.

The possibility to characterize DHP as electron-donating compounds, including interaction with ROS, was studied by determining their electrooxidation potentials [27]. Electrochemistry as a tool for studying antioxidant properties was proposed [28]. Recently Sürücü et al. [29] and Elkhouly [30] demonstrated good antioxidant/antiradical activity (by quenching superoxide radical) of some bicyclic DHP derivatives—4-aryl-2,6,6-trimethyl-5-oxo-1,4,5,6,7,8-hexahydroquinoline-3-carboxylates.

## 2. Materials and Methods

### 2.1. Materials

A set of six new 4-pyridinio 1,4-DHP compounds (see Table 1) was synthesized according to previously published methods (Duburs et al. [24] and Stonans et al. [25]).

A Fluo-4 NW Calcium Assay Kit was purchased from Invitrogen (Sweden). All other reagents were purchased from Sigma-Aldrich.

### 2.2. Cell Culture

The SH-SY5Y human neuroblastoma cell line (ATCC®, CRL-2266) and A7R5 aorta vascular smooth muscle cell line (ATCC®, CRL-1444) were obtained from LGC Standards AB (Sweden, European Collection of Animal Cell Cultures).

The SH-SY5Y human neuroblastoma and A7R5 aorta vascular smooth muscle cells were grown in Dulbecco's modified Eagle medium (DMEM) containing 1% nonessential amino acids and 2 mM glutamine and supplemented with 10% fetal bovine serum (FBS) at 37°C in a humidified atmosphere (incubator) with 5% CO_2_/95% air. The cells were passaged once a week using 0.25% trypsin and 0.53 mM EDTA solution and grown in 75 mm^2^ plastic culture flasks until confluent (then seeded onto 96-well plates for experiments). The cells were plated into a 96-well plate at 30,000 cells per well and incubated for 24 hours in DMEM with 10% FBS.

### 2.3. Intracellular Ca^2+^ Measurements

The intracellular free Ca^2+^ concentration [Ca^2+^]_i_ was measured in confluent monolayers of SH-SY5Y or A7R5 cells with the Ca^2+^-sensitive fluorescent indicator Fluo-4 NW according to the instruction (Fluo-4 NW Calcium Assay Kit, Thermo Fisher Scientific, cat. No. F36206) (as referenced by Vilskersts et al. [20].

The investigation of Ca^2+^ channel blocking activity of DHP derivatives was based on the effect of DHP on carbachol evoked intracellular Ca^2+^ mobilization in human neuroblastoma cells.

Carbachol affects calcium fluxes above all being cholinergic intracellular calcium regulator in neuroblastoma SH-SY5Y cells, as well as in aorta cell line A7R5. Its pronounced calcium agonist activity (even in the presence of potential calcium antagonist DHP compounds) is shown in [21, 22].

The cells were preincubated in the dark for 15 minutes with the tested compounds at concentrations ranging from 0.16 to 100 *μ*M. The application of carbachol (100 nM) to Fura-4 NW loaded SH-SY5Y cells stimulated a classic “biphasic” response. The well-known 3^rd^ generation calcium channel inhibitor amlodipine, which is also claimed to act as antioxidant and free radical scavenger [31], was used as the positive control (at the concentration range from 20 to 100 *μ*M).

For the investigation of Ca^2+^ channel agonist activity of the DHP compound, 4 Fura-4 NW loaded SH-SY5Y or A7R5 cells were stimulated by the addition of compound 4 at the concentration range from 4 to 100 *μ*M. The well-known calcium channel agonist Bay K8644 was used as the positive control (at the concentration range from 4 to 100 *μ*M).

Changes in [Ca^2+^]_i_ were measured from the fluorescence emitted at 516 nm due to alternate excitation at 494 nm using the microplate reader Tecan Infinite M1000 (Austria). Intracellular Ca^2+^ concentration was calculated from a standard curve with known amounts of free Ca^2+^ using the standard Ca^2+^-EGTA buffering system (Calcium Calibration Buffer Kit, cat. No.C-3008, Thermo Fisher Scientific).

The IC_50_ values for the tested compounds were calculated using GraphPad Prism 4.0 software.

In some experiments, Fura-4 NW loaded cells were washed and placed in normal Ca^2+^-free HEPES-buffered replacement medium. In Ca^2+^ restoration experiments, Ca^2+^ was initially absent from the medium and was restored to 1 mM after 870 s of stimulation with compound 4.

Where indicated, the cells were preincubated with inhibitors of Ca^2+^ signaling proteins (amlodipine, nifedipine, nicardipine, mecamylamine, atropine, or 2-aminoethoxydiphenyl borate (2-APB) in dose 100 *μ*M; procaine in dose 10 mM for 15 min, or with 5 *μ*M thapsigargin for 10 min, or with 100 *μ*M ruthenium red for 5 min), followed by stimulation with 100 *μ*M of compound 4. The commercial calcium agonist compound Bay K8644 was used as the reference compound.

### 2.4. Isolated Rat Aortic Ring Experiment

The experimental animal (one rat) was anesthetized with pentobarbital sodium (60 mg/kg i.p.). Once deep anesthesia was achieved, the thoracic aorta was dissected, placed in ice cold Krebs-Henseleit (K-H) buffer (content in mM: NaCl 118, KCl 4.7, CaCl_2_ 2.5, MgCl_2_ 1.64, NaHCO_3_ 24.88, KH_2_PO_4_ 1.18, glucose 10.0, sodium pyruvate 5.0, and disodium salt of ethylenediaminetetraacetic acid 0.05), cleaned from connective and fatty tissues, and cut into 3-4 mm long rings. Endothelium was mechanically scraped off with the tips of forceps, and the aortic rings were mounted between two stainless steel hooks attached to an isometric force transducer in 20 ml organ bath in K-H buffer bubbled with 95% O_2_ and 5% CO_2_ at 37°C (one separate aortic ring was used for each tested compound). Passive tension was adjusted to approximately 40 mN over a 60 min equilibration period. The experiment was started after the equilibration period by adding KCl solution to obtain a concentration of 50 mM. Addition of KCl induced contraction of the aortic rings, and a plateau of contraction was reached about 80 mM concentration. Thereafter, the tested compound solutions in dimethyl sulfoxide were added to the aortic rings in order to obtain the final concentrations in the range of 10^−10^-10^−5^ M. Changes in the tension were recorded using LabChart Pro software from ADInstruments (https://www.adinstruments.com/products/labchart/labchart-for-research). The EC_50_ values of the tested compounds were calculated using Prism 3.0 software from GraphPad (GraphPad Software Inc., USA) (https://www.graphpad.com/scientific-software/prism/) [20]. The experimental procedures involving experimental animals were carried out in accordance with the guidelines of the European Community, LR local laws and policies, and were approved by the Latvian Animal Protection Ethical Committee, the Food and Veterinary Service, Riga, Latvia.

### 2.5. Statistical Analysis

Comparisons between different groups were performed using Student's two-tailed unpaired *t*-test. *P* < 0.05 was considered to be a significant difference. All values are given as the mean ± SD.

### 2.6. Experiments for Evaluating the Electrochemical Oxidation Potentials of DHP

The cyclic voltammetry experiments were carried out on the PARSTAT 2273 electrochemical system. A stationary glassy carbon disk electrode (*d* = 0.8 mm) served as the working electrode, while the counter electrode was a Pt wire. The oxidation potentials were measured relative to Ag/Ag^+^ reference electrode. Acetonitrile was dried over P_2_O_5_ and distilled; the distillate was stored over CaH_2_ and redistilled just before use. Recrystallized tetrabutylammonium tetrafluoroborate (TBABF4) was used as a supporting electrolyte at 0.1 M concentration.

## 3. Results

### 3.1. Chemistry

#### 3.1.1. The Electrooxidation Potentials of DHP

The electrooxidation potential of 1,4-DHP compound 6 was 1.78 V, slightly higher than that for the analogue containing 4-phenyl group (1.74 V). Perhaps, the steric effect of 3,5-ethoxycarbonyl-methoxycarbonyl groups affected the electrooxidation potential. One could expect an analogous shift for DHP containing bulkier substituents at the positions 3 and 5.

Compound 2 has electrooxidation potential at 1.69 V, compounds 3 and 4 have potentials at 1.68 V, and compound 5 has electrooxidation potential at 1.70 V.

Antioxidant activity of quite a lot of 4-aryl-1,4-DHP (including 4-nitrophenyl-1,4-DHP comprising electron withdrawing nitrophenyl moiety as analogue to the pyridinio group) is reviewed in [1].

### 3.2. The Calcium Antagonist and Agonist Effects of DHP Derivatives on SH-SY5Y Human Neuroblastoma Cells and A7R5 Aorta Smooth Muscle Cells

#### 3.2.1. The Calcium Antagonist and Agonist Effects of DHP Derivatives on SH-SY5Y Human Neuroblastoma Cells

The Ca^2+^ channel agonist activity of the original DHP compounds (Table 1) was assayed *via* measuring the changes of intracellular calcium ion concentration [Ca^2+^]_i_ in the SH-SY5Y human neuroblastoma cells. The antagonistic action of DHP compounds was tested by observing the changes in carbachol-dependent mobilization of intracellular Ca^2+^ in the SH-SY5Y cells. The results are summarized in Table 2.

As shown in Table 2, compound 4 was the most potent [Ca^2+^]_i_ agonist with at least 9.2-fold higher activity than the well-known calcium channel agonist Bay K8644 (as the reference drug). On the contrary, the antagonist activity of compound 6 is approximately nine times lower than that of the 3^rd^ generation calcium antagonist amlodipine [31]. Ca^2+^ ion channel antagonist activity is maximal in the case of N-phenacyl derivative 3 (IC_50_ = 3.6 *μ*M); it even is superior comparing to amlodipine (approximately three times).

Compound 4 showed a dual effect: on one hand, it had antagonist properties comparable to amlodipine, but on the other hand, it also had at least 9.2 times stronger agonist activity than the well-known calcium channel agonist Bay K8644.

The Ca^2+^ ion channel antagonist activity was the highest in the case of the *N*-phenacyl derivative 3 (IC_50_ = 3.6 *μ*M), which was even approximately 3 times more active than amlodipine. The activity diminished in the case of a *p*-benzyloxy substituent in the phenyl group, and an additional methyl substituent bonded to the active methylene group of the phenacyl moiety (12 *μ*M, compound 4), but the activity was still equivalent to that of amlodipine. The antagonist activity was significantly diminished in the case of *p*-methoxy or *p*-nitro groups bonded to the phenacyl moiety (compounds 1 and 4), and there was no activity in the case of aliphatic *N*-ethoxycarbonylethyl moiety (compound 2) or the 4-(1,4-DHP)-pyridinio compound 5.

**Scheme 1:**
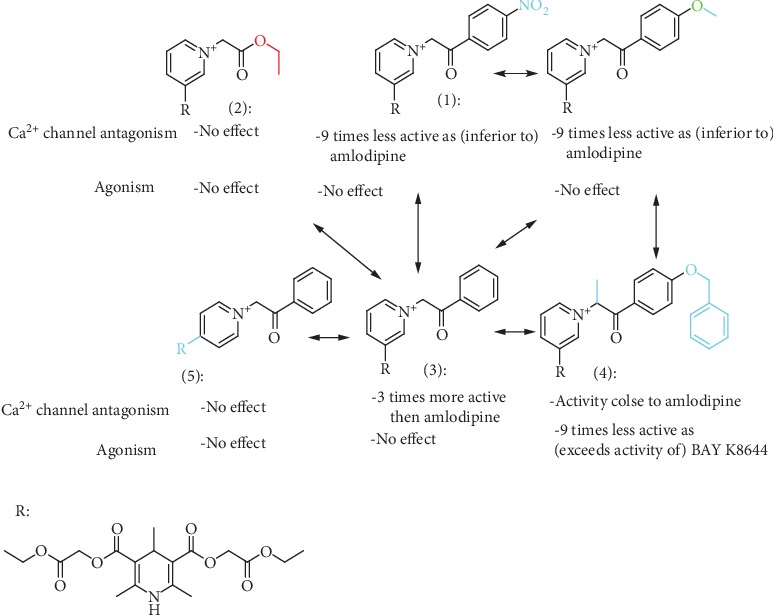
Structure-activity dependence of studied 1,4-dihydropyridines.

The structure of the 4-pyridinio moiety in the studied compounds determined the Ca^2+^ ion channel antagonist or agonist activity (for details of structure-activity relationship analysis, see Scheme 1, in Discussion).

#### 3.2.2. Concentration Dependence of the Agonist Effect for Compound 4

**Figure 1:**
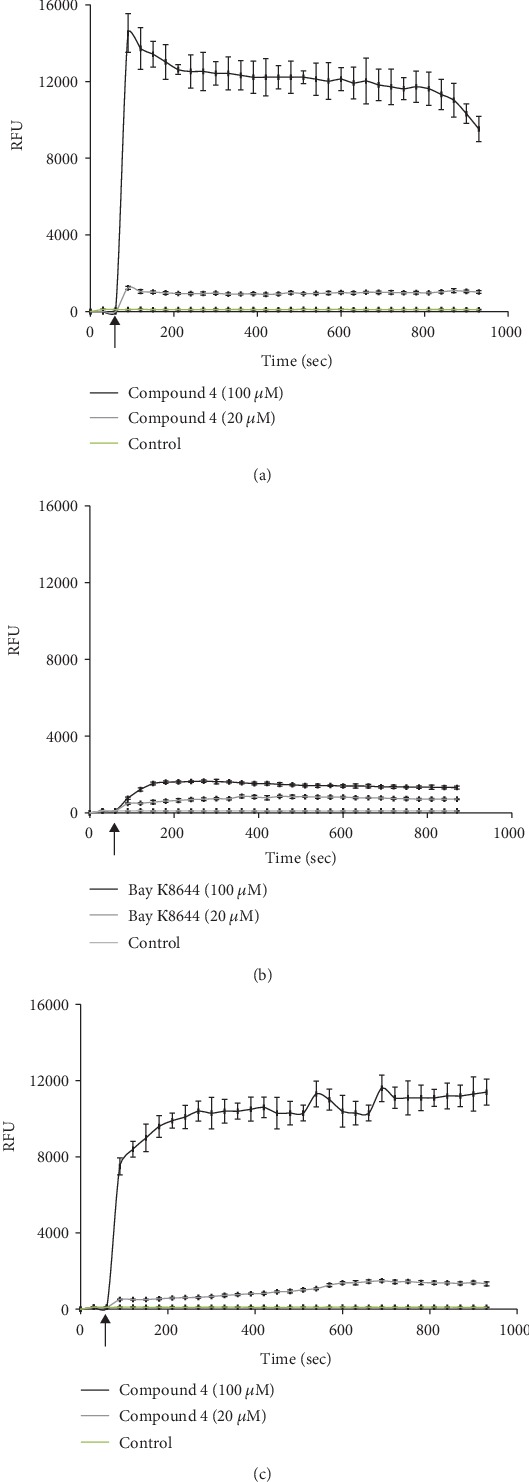
The dose-dependent calcium ion channel agonist activity. (a) [Ca^2+^]_i_ responses to different concentrations of agonist compound 4 in SH-SY5Y cells. (b) [Ca^2+^]_i_ responses to different concentrations of agonist Вау K8644 in SH-SY5Y cells. (c) [Ca^2+^]_i_ responses to different concentrations of agonist compound 4 in A7R5 cells. The cells were stimulated by the addition of compound 4 or Bay K8644 in concentration range from 20 to 100 *μ*M. Control is Fura-4 NW loaded cells without stimulator. Arrow indicates the time of compound 4 and Bay K8644 addition. Values were presented in RFU (relative fluorescence units) ± SD.

Compound 4 showed dose-dependent Ca^2+^ ion channel agonist activity on the SH-SY5Y neuroblastoma cells and A7R5 aorta smooth muscle cells (Figure 1) and caused a two-phase response similar to Bay K8644 on the SH-SY5Y cells.

In the studies on the A7R5 aorta cell line, compound 4 showed a dose-dependent agonist activity, evoking a two-phase response (see Figure 1(c)). It is important that 100 *μ*M concentration of compound 4 has substantially higher effect. After reaching the peak level, the oscillatory mode of Ca^2+^ ion response is remarkable, about one oscillation in 200 s.

In our article, it was accented the effect of compound 4 in general on the neuroblastoma cells. Therefore, Bay K8644 effect was tested only on SH-SY5Y cells. On A7R5, cells were estimated compound 4 effect too. The results about the effect of Bay K8644 on A7R5 cells are available in scientific article [21].

#### 3.2.3. Dependence of the Ca^2+^ Ion Response to Compound 4 on Presence or Absence of Extracellular Ca^2+^

**Figure 2:**
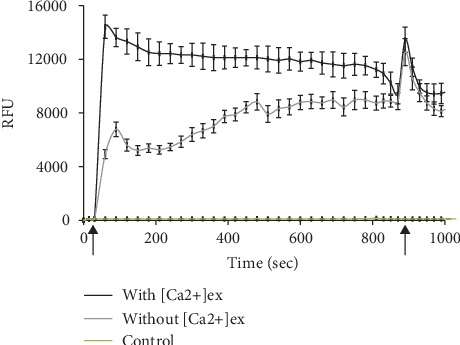
Sources of compound 4-mediated [Ca^2+^]_i_ signals in SH-SY5Y cells. The cells were stimulated with 100 *μ*M compound 4 in the presence or absence of extracellular Ca^2+^ ions in medium. Typical traces of [Ca^2+^]_i_ responses in the presence (black) or absence (grey) of 1 mM Ca^2+^. The first arrow indicates the time of compound 4 addition. Subsequent addition of 1 mM Ca^2+^ as indicated (the second arrow) 870 s after stimulation with compound 4. Control is RFU level in Fura-4 NW loaded cells without stimulator. Values were presented in RFU (relative fluorescence values/relative fluorescence units) ± SD.

The effect of compound 4 at 100 *μ*M concentration on the Ca^2+^ response was examined in the presence or absence of extracellular Ca^2+^ ([Ca^2+^]_ex_). In the absence of extracellular Ca^2+^, the level of intracellular [Ca^2+^]_i_ signal due to the compound 4 was decreased by 50% compared to that in the presence of extracellular Ca^2+^. This was confirmed by an increase of intracellular Ca^2+^ ion concentration when Ca^2+^ ions were added at 1 mM concentration to the Ca^2+^-free buffer after stimulation with compound 4. If the experiments were performed in a medium lacking Ca^2+^ ions, the level of the released Ca^2+^ ions decreased by 50%. A subsequent addition of Ca^2+^ ions at 1 mM concentration for 870 sec caused a peak of Ca^2+^ channel activation (Figure 2). This observation suggested that the action of compound 4 involved not only the liberation of Ca^2+^ ions from intracellular stores but also entry of Ca^2+^ ions from outside of the cell (Figure 2).

#### 3.2.4. The Effects of L-Type Calcium Channel Inhibitors Amlodipine, Nifedipine, and Nicardipine, as well as Mecamylamine and Atropine, on the Agonist Effect of Compound 4

Main two Ca^2+^ ion channel proteins include the dihydropyridine receptor (DHPR), normally a voltage-dependent calcium channel (VOC), as well as close structurally situated ryanodine receptors (RyRs). Besides, receptor-operated calcium channels (ROC) may regulate calcium ion influx and efflux.

To identify the source of Ca^2+^ ion influx and calcium ions channel types induced by compound 4, we studied the effect of known antagonists of Ca^2+^ channels: (1) the classical L-type calcium channel inhibitors—DHP derivatives nifedipine, nicardipine, and amlodipine; (2) atropine, a muscarinic receptor antagonist; (3) mecamylamine, a nonselective and noncompetitive nicotinic receptors blocker.

**Figure 3:**
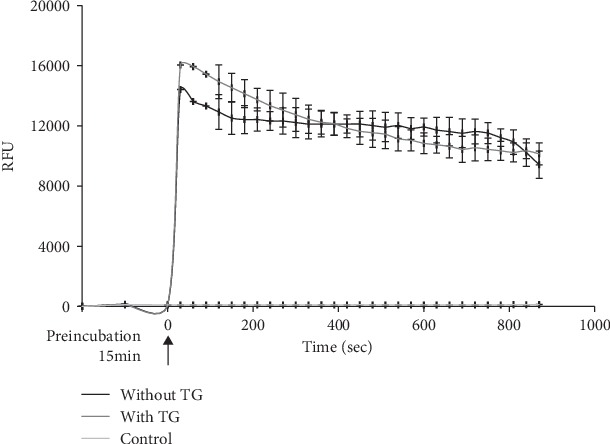
Role of SERCA in compound 4-induced [Ca^2+^]_i_ increase. Changes in [Ca^2+^]_i_ were monitored after pretreatment with thapsigargin (TG), followed by addition of 100 *μ*M compound 4 in Ca^2+^-free medium. Typical [Ca^2+^]_i_ response to compound 4 after pretreatment of cells with vehicle (black) or with 5 *μ*M thapsigargin (grey) for 15 min. Arrow indicates the time of compound 4 addition. Control is Fura-4 NW loaded cells without stimulator. Values were presented in RFU (relative fluorescence units) ± SD.

Pretreatment of the cells with 100 *μ*M of amlodipine, nifedipine, or nicardipine inhibited the [Ca^2+^]_i_ influx mediated by compound 4 by 50%, 42%, or 33%, respectively. Incomplete inhibition indicated that the L-type Ca^2+^ channels were only partially responsible for [Ca^2+^]_i_ level changes in this cell line (Table 3). As shown in Figure 3, amlodipine inhibited the first phase of the response, while nifedipine inhibited both phases.

**Figure 4:**
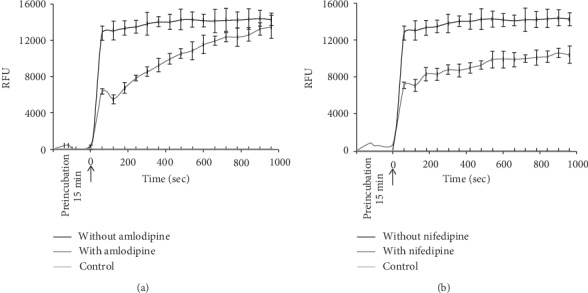
The effect of L-type calcium ion channel antagonists on evoked by compound Ca^2+^ ion channel activity. (a) The effect of amlodipine on evoked by compound 4 Ca^2+^ ion channel activity. (b) The effect of nifedipine on evoked by compound 4 Ca^2+^ ion channel activity. Fura-4 NW loaded SH-SY5Y cells were preincubated with amlodipine and nifedipine in the 100 *μ*M concentration for 15 min, and thereafter, the cells were stimulated (arrow) by the addition of compound 4 (concentration 100 *μ*M). Control is Fura-4 NW loaded cells without stimulator. Values were presented in RFU (relative fluorescence units) ± SD.

To identify the source of Ca^2+^ ion influx induced by compound 4, we studied the effect of known Ca^2+^ channel antagonists: (1) the structurally different classic L-type Ca^2+^ channel inhibitors—DHP derivatives nifedipine and amlodipine (the results are shown in Figure 4) and for nifedipine, nicardipine, and amlodipine summarized in Table 3; (2) atropine, a nonselective muscarinic receptor antagonist; and (3) mecamylamine, a nonselective and noncompetitive nicotinic receptor blocker. Pretreatment of cells with 100 *μ*M of nifedipine, nicardipine, or amlodipine inhibited the agonist response evoked by compound 4 by 42%, 33%, or 50%, respectively.

Incomplete inhibition indicated that the L-type Ca^2+^ channels were only partially responsible for the Ca^2+^ ion influx in this cell line (see Table 3).

As it is shown in Figure 4, amlodipine inhibited the first phase of the response, while nifedipine inhibited both phases.

The effect of amlodipine on the stimulation of calcium ion entry by compound 4 was different as shown in the literature [32]; where amlodipine antagonist effect IC_50_ 13 *μ*M is mentioned (on SH-SY5Y cells stimulated by 100 nM carbachol).

Mecamylamine (at concentrations from 4 to 100 *μ*M) had no effect on Ca^2+^ ion channel activity in the presence of compound 4; thus, nicotinic acetylcholine receptors were not involved in the response.

Atropine (at concentrations from 4 to 100 *μ*M) had no effect on Ca^2+^ ion channel activity in the presence of compound 4; therefore, this compound did not affect muscarinic receptors.

#### 3.2.5. The Role of ER Ca^2+^ Ion Increase Induced by Compound 4

To explore the role of sarco-/endoplasmic reticulum Ca^2+^- (SERCA-) ATPase (membrane transport protein ubiquitously found in the endoplasmic reticulum (ER) of all eukaryotic cells) in the mobilization of Ca^2+^ ions ([Ca^2+^]_i_) from sarco-endoplasmic reticulum in SH-SY5Y neuroblastoma cells treated with compound 4, we measured the increase of Ca^2+^ ion ([Ca^2+^]_i_) concentration for cells placed in medium free of Ca^2+^ ions. As shown in Figure 3, the noncompetitive inhibitor thapsigargin (5 *μ*M) of SERCA, which depletes Ca^2+^ stores in ER, had no influence on Ca^2+^ ion increase induced by compound 4. This indicated that mobilization of intracellular Ca^2+^ stores in SH-SY5Y neuroblastoma cells induced by compound 4 proceeded without involvement of SERCA.

Here, the main thesis—results in both cases (with and without TG)—are practically the same.

#### 3.2.6. The Independence of Agonist Effect due to Compound 4 from the Activation of Inositol 1,4,5-Trisphosphate Receptor (IP3R)

We also investigated the possible involvement of Gi/o/Gq/11 G protein-phospholipase C-IP3 receptor pathway in the [Ca^2+^]_i_ increase in neuroblastoma cells induced by compound 4. Pretreatment of cells with the cell-permeant IP3R inhibitor 2-APB for 15 min had no effect on the Ca^2+^ channel response induced by compound 4.

#### 3.2.7. The Role of Ryanodine Receptor (RyR) in Ca^2+^ Response Induced by Compound 4

The role of RyRs, another class of intracellular Ca^2+^ releasing channels, was subsequently tested with respect to the Ca^2+^ response in SH-SY5Y neuroblastoma cells induced by compound 4. As before, the experiments were conducted in Ca^2+^-free medium to minimize the interference from Ca^2+^ influx into the cells. Ruthenium red, a potent RyR inhibitor, inhibited the Ca^2+^ rise induced by compound 4 in a dose-dependent manner (Table 4). When the cells were pretreated for 15 min. with procaine, another well-known inhibitor of RyR, the Ca^2+^ rise induced by compound 4 was also inhibited in a dose-dependent manner (Table 4).

After preincubation with ruthenium red, the Ca^2+^ response to compound 4 is different in case of 50 and 100 *μ*M. After preincubation with procaine, results are close together in case without procaine and 5 mM procaine. In the case of preincubation with 100 *μ*M ruthenium red or 10 mM procaine, the Ca^2+^ response induced by compound 4 was absent.

#### 3.2.8. The Effect of Carbachol on the Activity of Compound 4

**Figure 5:**
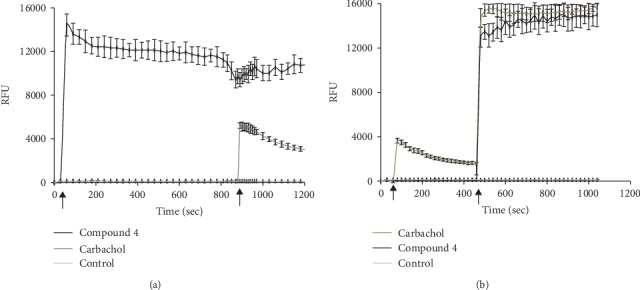
Carbachol effect on agonist activity of compound 4. (a) The carbachol-induced [Ca^2+^]_i_ increase after stimulation of cells with compound 4. The cells were stimulated with compound 4 (first arrow) and after 880 s were stimulated with 100 nM carbachol (second arrow). (b) The compound 4 induced [Ca^2+^]_i_ increase after stimulation of cells with carbachol. The cells were stimulated with 100 nM carbachol (first arrow) and after 460 s were stimulated with 100 *μ*M compound 4 (second arrow). Control is Fura-4 NW loaded cells without stimulator. Values were presented in RFU (relative fluorescence units) ± SD.

The addition of carbachol (100 nM) 880 s after the stimulation with 100 *μ*M of compound 4 did not cause stimulation of calcium channels. The addition of carbachol at 20 and 4 *μ*M concentrations caused activation that was weaker than that in the control experiment. On the other hand, the addition of compound 4 (100 *μ*M) 460 s after the stimulation with carbachol (100 nM) caused an activation peak comparable to the control experiment—stimulation only with compound 4 (Figure 5(b)).

It is possible that compound 4 inhibited the effect of carbachol as antagonist, or during the activation with compound 4 and carbachol, the same calcium stores were released, and the activation mechanisms were the same or partially similar (Figure 5 and Table 5). However, the effect of compound 4 did not diminish after the activation with carbachol; thus, it is possible that the mechanisms do not overlap and compound 4 can be recognized as calcium channel antagonist.

After reaching the peak level (Figure 5(a)) there, one can check (till 1200 s—the end of real registration time) Ca^2+^ ion oscillations, about one oscillation cycle in 50 s. In the case of compound 4, oscillations were more expressed as in the case of carbachol. It took time to check the mode of oscillations before the next agent could be added.

Carbachol is used and serves as the control (reference) compound—it alone leads to increase of fluorescence (Figure 5(b)). The effect of compound 4 (before carbachol addition, Figure 5(a)) on [Ca^2+^]_i_ increases is expressed more effectively than that of carbachol alone.

#### 3.2.9. The Effect of Compound 4 on Calcium Channel Activity in the A7R5 Aorta Cell Line

In the studies on the A7R5 aorta cell line, compound 4 showed a dose-dependent agonist activity, evoking a two-phase response (see Figure 1(c)). It is important that 100 *μ*M concentration of compound 4 has substantially higher effect. After reaching the peak level, the oscillatory mode of Ca^2+^ ion response is remarkable, about one oscillation in 200 s.

#### 3.2.10. Experiments with Isolated Rat Aortic Rings

The calcium channel antagonist activity of compound 4 was evaluated in potassium chloride precontracted denuded rat aortic rings. Denuded aortic rings were selected for this purpose to exclude any direct effects of the tested compound on the endothelium, taking into account that some authors have shown a release of vasodilator substances from the endothelium in the presence of 1,4-DHP derivatives [33].

Amlodipine, a well-known calcium channel inhibitor, was used as a positive control. The obtained results are summarized in Table 6. Compound 4 exhibited calcium channel blocking activity in the isolated rat aortic ring model, which was about 50 times weaker than the effect of amlodipine and significantly less expressed than in the SH-SY5Y cell line.

Compound 4 did not cause contractions of aorta rings; thus, there was no agonist effect in this model.

In the case of the aorta model, the studied DHP derivative 4 most likely showed antagonism against the Ca^2+^ V 1.2 channel subtype.

## 4. Discussion

Both Ca^2+^ channel antagonist and agonist effects on mainly SH-SY5Y neuroblastoma cells were revealed using fluorescence counting data in a group of six compounds that were derived from 4-pyridinio-1,4-dihydropyridines.

The manifestation and expression of calcium antagonist/agonist activity depends on the substituent structure of studied 1,4-DHP compounds (see Scheme 1).

The obtained data revealed that two of the mentioned derivatives (compounds 2 and 5) lacked any activity, while the remaining four derivatives showed Ca^2+^ antagonist properties of various degrees. One derivative (compound 4) could be characterized as Ca^2+^ channel agonist and antagonist.

A remarkable effect of 4-pyridinio moiety of studied compounds on Ca^2+^ ion channel antagonist or agonist activity was observed (see Scheme 1).

As shown (Table 2) and mentioned in Results, Ca^2+^ ion channel antagonist activity is maximal in the case of N-phenacyl derivative 3 (IC_50_ = 3.6 *μ*M); it even is superior comparing to amlodipine (approximately 3 times). Activity is diminished as a result of *p*-benzyloxy substituent in the phenyl group and insertion of methyl radical in the active methylene group of the phenacyl (IC_50_ = 12 *μ*M, compound 4); still, activity is similar to that of amlodipine. Antagonist activity is significantly diminished in the case of *p*-methoxy or *p*-nitro group at phenacyl moiety (compounds 1 and 4), and it is absent in case of aliphatic N-ethoxycarbonylethyl moiety (compound 2) or transition to isomeric 4-1,4-DHP-pyridinio compound 5. We could observe Ca^2+^ ion channel agonist activity only in the case of compound 4, so the 4-benzyloxy group and methyl radical in the active methylene groups are beneficial.

The rise of [Ca^2+^]_i_ evoked by compound 4 showed a biphasic effect that was comparable to that of the well-known Ca^2+^ channel agonist Bay K8644. Therefore, by stimulation of the cells using compound 4, a rise of [Ca^2+^]_i_ occurred not only from the intracellular stores of Ca^2+^ but also due to influx of Ca^2+^ into the cell from the extracellular medium. This was confirmed by the 50% decrease of the response in the absence of extracellular calcium ions in the incubation medium.

The fact that the rise of [Ca^2+^]_i_ due to compound 4 was inhibited by two different antagonists of the dihydropyridine receptors (DHPRs)—nifedipine that binds to specific sites on the DHPRs, known as dihydropyridine sites (see Copello et al. [34]), and the 3^rd^ generation Ca^2+^ antagonist amlodipine—suggests that compound 4 activated the dihydropyridine receptor of L-type Ca^2+^ channels (DHPRs) similar to the action of Вау K8644.

Thus, the Ca^2+^ agonist properties of compound 4 were very similar to that of Bay K8644, but compound 4 also had Ca^2+^ antagonist properties. Bay K8644 lacked these properties in our experiment.

The lack of the effect from atropine and mecamylamine on the rise of [Ca^2+^]_i_ evoked by compound 4 suggested that this compound had no effect on the muscarinic and nicotinic receptors.

The inhibition of sarco-/endoplasmic reticulum Ca^2+^- (SERCA-) ATPase by thapsigargin had no effect on the rise of [Ca^2+^]_i_ evoked by compound 4. This confirmed that compound 4 has no effect on the SERCA activity. These observations agree with the conclusions about Ca^2+^ modulator activity by DHP derivatives published by Copello et al. [34].

Two families of calcium-release channels have been extensively characterized, the RyRs and the inositol 1,4,5-triphosphate receptors (IP3R). Although RyRs are the major calcium release channels in striated muscle, IP3R are also present in smaller amounts and both types of channels occur in many other types of mammalian cells [35].

However, in our studies, the Ca^2+^ rise in cells incubated in Ca^2+^-free medium in the presence of compound 4 was not blocked by the IP3R inhibitor 2-APB, suggesting that this effect of compound 4 was not mediated by the activation of IP3Rs in this cell line.

Consistently with these results, the pretreatment of cells with either 30 *μ*M ruthenium red or 10 mM procaine, both of which are specific inhibitors of the RyRs, completely blocked the Ca^2+^ rise induced by compound 4. Therefore, we concluded that the Ca^2+^ rise from Ca^2+^ stores, as induced by compound 4, could proceed through the activation of RyRs in SH-SY5Y cells. Thus, compound 4 affected Ca^2+^ ion entry through dihydropyridine receptor L-type Ca^2+^ channels (DHPRs), activating the RyR Ca^2+^-release channels, but had no effect on SERCA-mediated Ca^2+^ uptake. These findings are in context with the data about Ca^2+^ modulator activity by DHP derivatives obtained by Copello et al. [34]. It has been previously shown that Вау K8644 did not affect the rate of Ca^2+^ uptake into SR microsomes [34].

The addition of carbachol after stimulation with compound 4 did not cause the activation of calcium channels. At lower doses of compound 4, a dose-dependent inhibition of Ca^2+^ channels activated by carbachol was observed; thus, the Ca^2+^ antagonist properties of compound 4 were demonstrated. On the other hand, the addition of compound 4 after activation with carbachol led to a rise of Ca^2+^ influx, which was comparable to the effect of compound 4 alone. This suggests that the mechanism of calcium channel activation in the case of compound 4 was different from that of carbachol. Therefore, compound 4 had simultaneously both antagonist and agonist properties. A dual effect of DHP has been reported [3, 10]. The agonist effect caused by Bay K8644 depended on its concentration, the membrane potential, and changes in channel composition. The antagonist properties of Bay K8644 were shown to induce important changes in the channel properties [9]. An analogous dependence on changes of the membrane potential has been reported for the calcium antagonist nitrendipine [7].

In the experiments with the A7R5 aorta cell line, it was also observed that compound 4 activated calcium channels. However, in studies using isolated rat aortic rings, only inhibition of calcium channel activity occurred and the contractions of the aortic rings were not affected; thus, there was no agonist effect. At the same time, Вау K8644 showed an agonist effect also in experiments with porcine coronary artery rings [12].

The chemical structure determinants and hydrophobic/hydrophilic properties of DHP derivatives that act as Ca^2+^ modulators have been widely studied. The antagonist and agonist activities are associated with different parts of the DHP molecules and have different mechanisms, as proposed by Tikhonov and Zhorov [36]. The spatial configuration of the DHP core structure allows accommodation of long substituents in the domain interface or in the inner pore of the LTCC channels. It was proposed that the hydrophilicity or hydrophobicity of the portside group at the DHP core structure provides for the antagonist or agonist character of DHP derivatives. Hydrophobic groups such as COOMe promote an antagonistic effect, whereas hydrophilic groups like NO_2_ promote an agonistic effect. Thus, agonists such as (*S*)-Bay K8644 bear an NO_2_ group at the portside. Other agonists also have a small hydrophilic substituent at the portside such as nitrile, lactone, and thiolactone moieties. Antagonists, on the contrary, have hydrophobic portside groups.

DHPs can also act as either antagonists or agonists, depending on the different experimental conditions and the structures of the drug targets [5, 7, 15, 37]. The effects of DHPs are Ca^2+^ dependent. It has been proposed that DHP antagonists bind to and stabilize a nonconducting channel state in which the selectivity filter is occupied by a single Ca^2+^ ion. The binding of a second Ca^2+^ ion is considered to destabilize the DHP binding [38–40]. As it has been shown for (-)-Bay K 8644, it resembles a racemic compound; it enhances or inhibits calcium ion currents depending on the holding potential. The results of this study suggest that the dual activity of the racemic compound is not because of the opposing effects of its component enantiomers [37]. In our studies, compound 4 was a racemic DHP. Small changes to the structure of LTCC (L-type calcium channels), as revealed by the behaviors of DHP in chimeric and mutagenized LTCC, can transform a DHP agonist into an antagonist (see Tikhonov and Zhorov [36]).

Despite the uncertainty discussed above, all six of our studied 4-pyridinio-1,4-DHP derivatives formally comply with the prediction [36] for the expression of Ca^2+^ agonist properties (namely, agonists should have either hydrophilic substituents or a hydrogen atom at the portside of DHP molecule and thus lack the destabilizing effect on Ca^2+^ binding to the selectivity filter glutamates, which is necessary for inducing long-lasting channel closure exerted by hydrophobic portside of antagonists). In reality, however, Ca^2+^ agonist activity was found only for compound 4.

Regarding structure-activity dependence of studied 1,4-dihydropyridines (see Scheme 1), the structure-activity relationships for the studied 1,4-DHPs give some observations about the expected impact of structural fragments on calcium antagonist/agonist activity expression.

The calcium agonist properties of compound 4 may be associated with the side chain moiety at the 4-pyridinio cycle, consisting of two benzene rings linked by a -OCH_2_- group and further connected to 4-pyridinio cycle by a -CH_2_CO- chain. If the moiety has simpler composition without this -OCH_2_- group and has only one benzene ring, the effect disappears. It is possible that this group is responsible also for the Ca^2+^ antagonist effect, because variations in this group caused changes in IC_50_. Compound 4 could be classified as a dual-acting antagonist and agonist simultaneously; moreover, these effects were observed on the same cell line. Calcium channel agonist properties were observed for compound 4 possessing ester groups at the positions 3 and 5 of the 1,4-dihydropyridine ring and lacking nitro or lactone groups at the positions 3 or 5. To the best of our knowledge, this is a novel observation. It has been mentioned that some 3,5-dipropargyloxycarbonyl-4-*N*-alkylpyridinio-1,4-DHPs possess calcium antagonist activity, and also, calcium agonist activity was detected (but not measured) on H9C2 and A7R5 cells [41].

In the present study, we have used the SH-SY5Y neuroblastoma cell line, as well as A7C5 aorta vascular smooth muscle cell line and isolated rat aorta rings, as targets for determining the effects of 4-pyridinio-1,4-dihydropyridines on Ca^2+^ ion transport. It is further planned to use cardiomyocyte cell cultures and/or cardiac tissues for elucidating the mode of action of compound 4, because it has been shown that the characteristics of the DHP derivatives, the tissue properties, and the types of stimuli are all relevant to the calcium channel modulation [42]. Our data coincide with the conception of [43] on calcium channel agonists and antagonist. Identical accommodation sites for nifedipine and Bay K8644 were postulated [43]. However, if the concentration of DHP agonists is high enough to overcome the energetic barrier, the dissociation of agonistic ligands and inactivated channel would be prevented; in that case, the agonists could function as antagonists [43]. The DHP derivatives possess 1,4-dihydropyridine scaffold, important for many physiological properties [5]. Many voltage-gated calcium ion channel blockers, comprising 1,4-DHP nucleus may possess pleiotropy-different (even more than listed 14) therapeutic effects, including antioxidant activity [44].

Compound 4 featuring a methyl-*p*-benzyloxyphenacyl moiety at the pyridinio ring nitrogen atom has double faced, Janus-type effect on neuroblastoma cells SH-SY5Y: Ca^2+^ channel agonist activity exceeding that of Bay K8644 9 times and Ca^2+^ channel antagonist activity comparable with that of amlodipine.

Concerning Janus-type compounds in literal meaning—e.g., fullerenols may comprise two types of substituents—each type on one half of the molecule [45]. In our case, we use the term “Janus-type compounds” as compounds causing two different biochemical or pharmacological effects, e.g., microtubule depolymerizing and stabilizing effects [46].

Electrooxidation data testify that 4-pyridinio compounds could possess antioxidant properties as proved for 4-aryl-DHP—calcium ion antagonists [1]. The relationship between antioxidant activity, first electrochemical oxidation potential, and spin population of flavonoid radicals is recently shown [47]. As reported in [48], used in the present study 4-Ph-DHP and commercial DHPs (4-Ph-DHP<nisoldipine<nifedipine<amlodipine<nimodipine) have high Ep ox and moderate AI values (relative antioxidant values) and their antioxidant effects may be attributed to the oxidation of DHP ring to the respective pyridine derivative. High antioxidant activities recently were found for some 4-aryl (4-chlorophenyl) 1,4-dihydropyridines [49].

## 5. Conclusions

In summary, we report calcium channel antagonist/agonist effect of novel series of 4-pyridinio-1,4-dihydropyridine (1,4-DHP) derivatives. For six compounds of this series calcium channel, modulating activity was evaluated using aorta cell line A7R5 cells, isolated rat aortic ring model, and SH-SY5Y human neuroblastoma cell line. A remarkable effect of 4-pyridinio moiety of studied compounds on Ca^2+^ ion channel antagonist or agonist activity was observed. Four of these compounds showed calcium channel blocking activity in SH-SY5Y, A7R5 cells, and the aortic ring model. The Ca^2+^ ion channel antagonist activity was the greatest in the case of the *N*-phenacyl derivative compound 3, 9 times exceeding activity of amlodipine, and agonist activity in case of compound 4, so 4-benzyloxy group and methyl radical in the active methylene groups are beneficial.

Compound 4 featuring a methyl-*p*-benzyloxyphenacyl moiety at the pyridinio ring nitrogen atom has double faced, Janus-type effect on neuroblastoma cells SH-SY5Y: Ca^2+^ channel agonist activity exceeding that of Bay K8644 9 times and Ca^2+^ channel antagonist activity comparable with that of amlodipine.

Concerning the mechanism of calcium ion channel activation by compound 4, it was revealed that the ryanodine receptors had the dominant role. Similar to the stimulation of cells using compound 4, the rise of [Ca^2+^]_i_ was not only due to release from the intracellular stores of calcium but also due to influx of Ca^2+^ ions into the cell from the extracellular medium.

Compound 4 did not cause the contractions of aortic rings; thus, there was no agonist effect in the case of this model. The performed structure-activity study enables better understanding of the interactions between 1,4-dihydropyridines and calcium channel. In addition, the studied compounds being DHP derivatives could serve as electron-donating entities for the prevention of oxidative stress.

A new therapeutic strategy, so called the multitarget small molecule (MTSM) approach, is based on the design of drugs able to bind simultaneously at diverse enzymatic systems or receptors involved in pathology [50]. 1,4-DHP bearing 2-pyridyl group at position 4 have both activator and antagonist properties, being cardiostimulant and vasorelaxant agents, so potential benefit for cardiac failure could be proposed [51].

Compound 4 has calcium channel agonist and antagonist properties on the same cell line SH-SY5Y.

The obtained data, especially the just mentioned peculiar property of compound 4, could be a basis for further studies to obtain similar compounds, to investigate structure-activity relationships of calcium transport modulation (directly or indirectly [52]), as well as receptor binding [53] of these 1,4-DHP derivatives, to understand better the molecular mechanism of activities and to explore the way to get tailor-made compounds possessing predicted properties.

## Figures and Tables

**Table 1:** A list of synthesized and studied six 1,4-DHP compounds, their numbers and structural formulas.

Compound no.	Structural formula
1	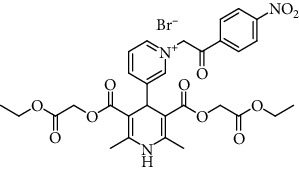
2	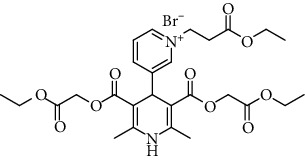
3	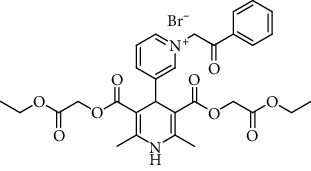
4	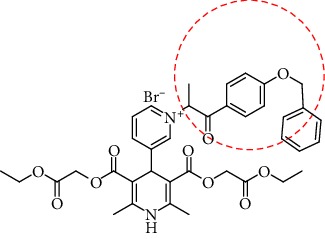
5	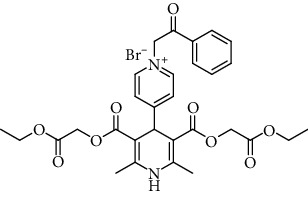
6	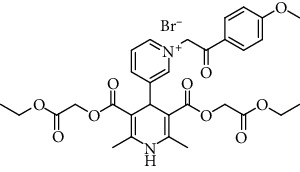

**Table 2:** The antagonist and agonist effects of 1,4-DHP compounds (1-6) on [Ca2+]_i_ in Fluo-4 NW-loaded SH-SY5Y cells. (Calcium antagonist amlodipine and agonist Bay K8644 were used as reference compounds).

Compound name and No. of studied 1,4-DHP	Antagonist activity (IC_50_, *μ*M)	Agonist activity (RFU, 100 *μ*M)
Amlodipine	11 ± 2	No effect
1	100 ± 12	No effect
2	No effect	No effect
3	3.6 ± 0.3	No effect
4	12 ± 2	14425 ± 1072
5	No effect	No effect
6	100 ± 9	No effect
Bay K8644	No effect	1553 ± 115

RFU: relative fluorescence units.

**Table 3:** The effect of L-type calcium ion channel antagonists amlodipine, nifedipine, and nicardipine on Ca^2+^ ion channel activity evoked by compound 4.

Concentration (*μ*M)	Inhibition (%)		
	Аmlodipine	Nifedipine	Nicardipine
100	50 ± 11	42 ± 5	33 ± 8
20	10 ± 11	16 ± 10	11 ± 5
4	0 ± 6	7 ± 8	10 ± 6
IC_50_ (*μ*M)	100	>100	>100

**Table 4:** The role of RyRs on Ca^2+^ response induced by compound 4.

Concentration (*μ*M)	Inhibition (%)	
	Ruthenium	Procaine
100	100 ± 4	98 ± 5
50	68 ± 8	18 ± 6
25	36 ± 8	0 ± 7
12.5	21 ± 10	5 ± 9
6.25	0 ± 9	0 ± 2
IC_50_ (*μ*M)	31 ± 8	61 ± 9

**Table 5:** The antagonist effect of compound 4 on Ca^2+^ channels activated with carbachol.

Concentration (*μ*M)	Inhibition (%)
20	71 ± 9
4	11 ± 12
0,8	4 ± 10
0,16	−16 ± 10
IC_50_ (*μ*M)	12 ± 2

**Table 6:** Calcium channel blocking activity in isolated rat aortic ring model (EC_50_) of the tested compounds.

Compound	EC_50_ (nM)
Amlodipine	14.3 ± 0.3
Compound 4	691 ± 167

## Data Availability

The research data used to support the findings of this study are included within the article (tables, figures).

## Abbreviations

DHP: 1,4-Dihydropyridine(s)
SH-SY5Y: Human neuroblastoma cell line
A7C5: Rat aorta cell line
FCS: Fetal calf serum
DMEM: Dulbecco's modified Eagle's medium
RyR: Ryanodine receptors
ER (SERCA): Sarco-/endoplasmic reticulum Ca^2+^-ATPase
2-APB: 2-Aminoethoxydiphenyl borate.
